# Serial measurements of cardiac biomarkers in patients after allogeneic hematopoietic stem cell transplantation

**DOI:** 10.1186/1756-9966-31-13

**Published:** 2012-02-09

**Authors:** Lubica Roziakova, Eva Bojtarova, Martin Mistrik, Juraj Dubrava, Jozef Gergel, Nadezda Lenkova, Beata Mladosievicova

**Affiliations:** 1 Institute of Pathological Physiology, School of Medicine, Comenius University, Bratislava, Slovakia; 2 Department of Hematology and Transfusion Medicine, University Hospital, Bratislava, Slovakia; 3 Non-invasive Cardiology Department, University Hospital Bratislava, Bratislava, Slovakia; 4 Department of Clinical Biochemistry, Medirex Bratislava, Bratislava, Slovakia

**Keywords:** Hematopoietic stem cell transplantation, Cardiotoxicity, Natriuretic peptides, Cardiac troponins

## Abstract

**Background:**

Previous therapy with anthracyclines (ANT) and conditioning regimen followed by hematopoietic stem cell transplantation (HSCT) represents a high risk for development of cardiotoxicity. The aim of this study was to assess subclinical myocardial damage after HSCT using echocardiography and cardiac biomarkers - high sensitive cardiac troponin T (hs-cTnT) and N-terminal pro-B-type natriuretic peptide (NT-proBNP) and to identify patients at risk of developing clinical cardiotoxicity.

**Patients and methods:**

Thirty-seven patients who were treated with allogeneic HSCT for hematologic diseases at median age of 28 years at time of HSCT were studied. Conditioning regimen included either chemotherapy without total body irradiation (TBI) or combination of chemotherapy with TBI. Twenty-nine (78,3%) patients were pretreated with ANT therapy. Cardiac biomarkers were serially measured before conditioning regimen and at days 1, 14 and 30 after HSCT. Cardiac systolic and diastolic functions were assessed before conditioning regimen and 1 month after HSCT by echocardiography.

**Results:**

The changes in plasma NT-proBNP and hs-cTnT levels during the 30 days following the HSCT were statistically significant (*P *< 0,01 v.s. *P *< 0,01). Persistent elevations of NT-proBNP and hs-cTnT simultaneously for a period exceeding 14 days after HSCT were found in 29,7% patients. Serum concentrations of cardiomarkers were significantly elevated in ANT group compared to non-ANT group. These observations were underscored by the echocardiographic studies which did reveal significant changes in systolic and diastolic parameters. Five of 37 (13,5%) patients developed clinical manifestation of cardiotoxicity.

**Conclusions:**

Elevations in both cardiac biomarkers were found before clinical signs of cardiotoxicity developed. Persistent elevations in NT-pro-BNP and hs-cTnT concentrations simultaneously for a period exceeding 14 days might be used for identification of patients at risk of developing cardiotoxicity and requiring further cardiological follow up.

## Background

Stem cells are widely used in the treatment of malignant and nonmalignant diseases [1]. Advances in allogeneic hematopoietic stem cell transplantation (HSCT) have increased survival in hematologic diseases. Among those who survive the first 2 years, nearly 80% of allogeneic HSCT recipients are expected to become long-term survivors and by 2020 there may be up to half a million of these survivors worldwide [2,3].

However, HSCT survivors are at risk of developing long-term complications. A fifth of HSCT survivors develop severe or life-threatening conditions [4]. Cardiac complications are frequently found life-threatening conditions. When cardiac dysfunction develops, complete recovery of cardiac function occurs in only 42% of patients, despite pharmacological therapy [5]. Hence, new approaches for early cardiotoxicity detection need to be validated widely. Measurement of cardiospecific biomarkers can be a valid diagnostic tool for early identification, assessment and monitoring of cardiotoxicity. This approach is minimally invasive, less expensive than echocardiography and easily repeated.

Cardiac biomarkers are routinely evaluated only in patients before HSCT with increased cardiac risk [6,7]. Future research should focus on the best timing for sampling, well-standardized methods for biomarkers determination and cut-off concentration that gives the best diagnostic accuracy in terms of sensitivity, specificity and predictive values. We therefore evaluated cardiotoxicity using cardiac biomarkers - high sensitive cardiac troponin T (hs-cTnT) and N-terminal pro-B-type natriuretic peptide (NT-proBNP) during HSCT in 37 patients in whom echocardiography was performed before and one month after HSCT.

## Patients and methods

### Patients

This prospective study involved 37 consecutive patients with a median age of 28 years (range: 19-58 years) who underwent an allogeneic hematopoietic stem cell transplantation (HSCT) from June 2009 to February 2011 at the Transplantation Centre of Hematology Department at University Hospital Bratislava. There were 24 males and 13 females. Their diagnosis included acute myeloid leukemia (AML) in 13 patients, acute lymphoblastic leukemia (ALL) in 14 patients, chronic myeloid leukemia (CML) in 2 patients, Hodgkin's lymhoma in one patient, myelodysplastic syndrome (MDS) in 3 patients, osteomyelofibrosis in one patient and severe aplastic anemia in 3 patients.

Twenty-seven patients were conditioned with myeloablative regimens including cyclophosphamide (CY) 60 mg/kg body weight intravenously on 2 consecutive days in combination with fractionated total body irradiation (TBI) 12 Gy in six fractions of 2 Gy over 3 days in 12 patiens or in combination with peroral busulphan 4 mg/kg body weight daily for 4 days in 15 patients. The remaining 10 patients were conditioned with nonmyeloablative regimens (cyclophosphamide, busulphan, fludarabine, etoposide, cytosine arabinoside, melphalan, idarubicin, carmustine or with combination of antithymocyte globulin). Fifteen patients received hematopoietic stem cells from an HLA-matched related donor and 22 patients from an HLA-matched unrelated donor.

Cyclosporine A and short-term methotrexate were administered for the prophylaxis of graft-versus-host disease (GVHD). Two patients had arterial hypertension, 2 patients had diabetes mellitus and 14 patients had dyslipidemia before transplantation. One patient had a prior history of a cardiac disease because of leukemic infiltration of the heart (at the time of diagnosis of acute leukemia). The cumulative dose of anthracyclines (ANT) (idarubicin, daunorubicin and mitoxantrone) was calculated as the equivalent dose of doxorubicin. Twenty-nine patients were previously treated with ANT (median 250 mg/m^2^, range: 100-470). Characteristics of patients are summarized in Table 1.

**Table 1:** Characteristics of patients

Age at HSCT (median)	19-58 (28 years)
**Sex, F/M**	13/24

**Diagnosis**	

AML	13

B-ALL	12

T-ALL	2

CML	2

SAA	3

HL	1

OMF	1

MDS	3

**Treatment before HSCT**	

ANT	29

cranial RT (24 Gy)	14

mediastinal RT (24 Gy)	2

**Conditioning regimen**	

BUCY2	15

TBI+CY	12

CY+ATG	1

BCNU+VP-16+ARAC+MEL	1

BU+FLU+CY	3

FLU+CY+ATG	2

BU+FLU+ATG	1

FLAMSA	2

*HSCT *hematopoietic stem cell transplantation, *AML *acute myeloid leukemia, *ALL *acute lymphoblastic leukemia, *CML *chronic myeloid leukemia, *SAA *severe aplastic anemia, *HL *Hodgkin's lymphoma, *OMF *osteomyelofibrosis, *MDS *myelodysplastic syndrome, *CY *cyclophosphamide, *BU *busulphan, *TBI *total body irradiation, *FLU *fludarabine, *ATG *antithymocyte globulin, *MEL *melphalan, *BCNU *carmustine, *VP-16 *etoposide, *ARAC *cytosine arabinoside, *FLAMSA *fludarabine + cytosine arabinosid + TBI + CY + amsacrine was replaced by idarubicin, *ANT *anthracyclines

The study was approved by the local ethics committee of University Hospital Bratislava. Written informed consent was obtained from all patients.

### Evaluation of cardiac function

Together 148 blood samples were evaluated in 37 patients. Serial measurements of plasma NT-proBNP and hs-cTnT concentrations were performed the day before conditioning regimen (baseline), the day after HSCT (D + 1), 14 days after HSCT (D + 14) and 30 days after HSCT (D + 30) in all patients. Venous blood samples were obtained from an indwelling catheter in the morning and serum concentrations of biomarkers were measured immediately by electrochemiluminescence immunoassay on Elecsys 2010 analyzer (Roche Diagnostics). The upper reference limit (99th percentil) for hs-cTnT was 0.014 μg/L and cut-off values for NT-proBNP excluding acute heart failure were 450 and 900 pg/mL for ages < 50 and 50-75 years [8,9].

Echocardiography was performed before the conditioning regimen and 1 month after HSCT. Parameters of systolic and diastolic left ventricular (LV) function were evaluated. Systolic LV dysfunction was defined as ejection fraction (EF) less than or equal to 50%. To evaluate LV diastolic function, the following parameters were recorded: peak flow velocity of early filling (E), peak flow velocity of late filling (A), ratio of peak early to peak late flow velocities (E/A), E-wave deceleration time (DT) and isovolumetric relaxation time (IVRT). Diastolic LV dysfunction was defined as E/A inversion and DT above 220 ms on the transmitral Doppler curve (impaired relaxation).

### Statistical analysis

Continuous variables (echocardiographic parameters) are presented as mean ± SD (standard deviation) and cardiac biomarkers (NT-proBNP, hs-cTnT) as median and interquartile range. Comparisons between continuous or categorical variables were performed using the Student's t-test, Mann-Whitney and Wilcoxon test. Friedman test was used to test the difference between variables. Correlations were evaluated with Spearman correlation coefficient. A *P*-value less than 0,05 was considered statistically significant.

## Results

**Figure 1:**
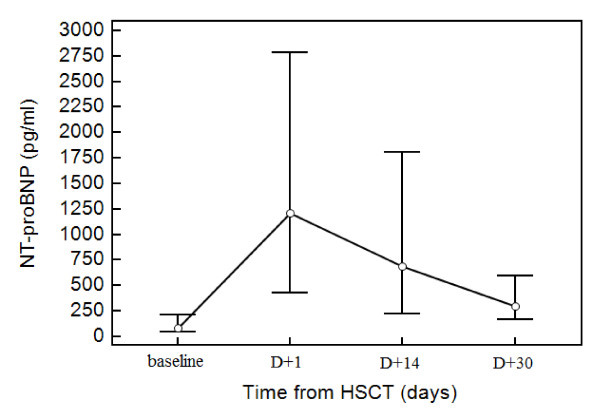
**The changes in plasma NT-proBNP level during HSCT**. The changes in plasma NT-proBNP level over the 30 days following the HSCT were statistically significant (*P *< 0,01). The highest values were detected on day 1 after HSCT in 26 (70,3%) patients with a gradual decline, but without normalization to baseline. Thirty days after HSCT, NT-proBNP remained elevated in 11 of 37 (29,7%) patients.

The changes in plasma NT-proBNP level during the 30 days following the HSCT were statistically significant (*P *< 0,01). The highest values were detected on day 1 after HSCT in 26 (70,3%) patients with a gradual decline, but without normalization to baseline (Figure 1). Fourteen days after HSCT, concentrations of NT-proBNP remained elevated in 23 of 37 (62,2%) patients and 30 days after HSCT in 11 of 37 (29,7%) patients. In patients who were previously treated with ANT, the NT-proBNP level in all measurements was significantly higher compared to those who were not treated with ANT (*P *= 0,01). There were no differences between patients with or without TBI as a part of conditioning regimen (*P *= 0,48). Levels of NT-proBNP showed no correlation with fever in the last week (ρ = 0,06; *P *= 0,4), with plasma creatinine level (ρ = 0,03; *P *= 0,7) and arterial hypertension (ρ = 0,02; *P *= 0,77).

**Figure 2:**
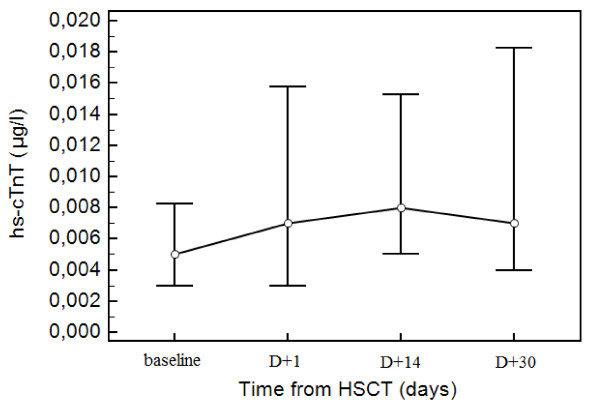
**The changes in plasma hs-cTnT level during HSCT**. The differences in plasma hs-cTnT level over the 30 days following HSCT were statistically significant (*P *< 0,01). Persistent elevations in hs-cTnT levels 1 day and also 30 days after HSCT were found in 27% vs 29,7% patients.

The differences in plasma hs-cTnT level during the 30 days following HSCT were also statistically significant (Figure 2, *P *< 0,01). We found persistent elevations in hs-cTnT levels 1 day, 14 days and also 30 days after HSCT (27% vs 29,7% vs 29,7% patients). The concentrations of hs-cTnT in all measurements were significantly higher in patients previously treated with ANT (*P *< 0,01), but not in patients receiving TBI as a part of the conditioning regimen (*P *= 0,14). Levels of hs-cTnT showed no correlation with fever in the last week (ρ = 0,02; *P *= 0,75), with plasma creatinine level (ρ = -0,02; *P *= 0,74) and arterial hypertension (ρ = -0,02; *P *= 0,78). Levels of NT-proBNP showed positive correlation with hs-cTnT (ρ = 0,35; *P *< 0,01).

In the early period after HSCT, we found a statistically significant decrease in systolic LV function (65 ± 5,7% at baseline, 61 ± 4,8% at 1 month; *P *< 0,01). The mean E/A ratio decreased significantly over time, whereas DT and IVRT remained unchanged (Table 2). Newly developed systolic dysfunction appeared in 5 (13,5%) patients and diastolic dysfunction in 2 (5,4%) patients. There were no differences in systolic echocardiographic parametres in patients previously treated with or without ANT and with or without TBI as a part of the conditioning regimen (*P *= 0,78 vs 0,27). Levels of NT-proBNP showed negative correlation with LV EF (ρ = -0,35, *P *= 0,03).

**Table 2:** Echocardiographic parameters before and after HSCT

	Before HSCT	After HSCT	*P*-value
**Systolic parameters**			

*LVEF (%)*	65 ± 5,7	61 ± 4,8	< 0,01

**Diastolic parameters**			

*E/A*	1,37 ± 0,22	1,07 ± 0,3	< 0,01

*DT (ms)*	174 ± 20,9	182 ± 24,5	0,3

*IVRT (ms)*	75,06 ± 7,5	79,11 ± 6,8	0,1

*LVEF *left ventricular ejection fraction, *A *peak flow velocity of late filling, *DT *E-wave deceleration time, *E *peak flow velocity of early filling, *IVRT *isovolumetric relaxation time

Of 37 patients, 5 (13,5%) developed a cardiac event. All of these patients exhibited elevated plasma NT-proBNP and hs-cTnT levels prior to clinical signs occuring and these elevations persisted at least 30 days after HSCT. Characteristics of patients are described in Table 3. In two patients, the cardiotoxicity was severe and resulting in death (one patient was diagnosed with pericarditis and another heart failure).

**Table 3:** Characteristics of patients with clinical cardiotoxicity

Patient	Clinical manifestation of cardiotoxicity	Day after HSCT	Baseline NT-proBNP/hs-cTnT	NT-proBNP/hs-cTnT	Conditioning regimen	CD ANT (mg/m^2^)
1	Chest pain, dyspnea	3	237/normal	9589/0,032	TBI + CY	390

2	Chest pain, dyspnea	1	320/normal	12 156/0,076	FLAMSA	125

3	Fluid retention, pericarditis	15	327/normal	3761/0,016	TBI + CY	150

4	Fluid retention	10	412/0,025	4817/ 0,047	BUCY2	470

5	Cardiogenic shock	176	63,88/0,018	31 444/0,05	TBI + CY	150

*ANT *anthracyclines, *CY *cyclophosphamide, *hs-cTnT *high sensitive cardiac troponin *T, NT-proBNP *N-terminal pro-B-type natriuretic peptide, *TBI *total body irradiation, *CD *cumulative dose, *FLAMSA *fludarabine + cytosine arabinosid + TBI + CY + amsacrine was replaced by idarubicin, *BU *busulphan

## Discussion

The results of this prospective and single-center study revealed, that persistently elevated cardiac biomarkers have important implications for identifying high-risk patients, particularly if levels of cardiac troponins and natriuretic peptides are simultaneously elevated for a period exceeding 14 days. We found that NT-proBNP and hs-cTnT might be a useful diagnostic tool for early detection of cardiotoxicity before its clinical manifestation. All patients with clinical cardiotoxicity had contemporary elevations in both cardiac biomarkers before clinical signs developed.

Natriuretic peptides elevations have been shown to reflect wall stress, and thus provide functional information. Although the usefulness of NT-proBNP is well known in detection of chemotherapy-induced cardiotoxicity, only a few reports have assessed the detection of cardiotoxicity using BNP/NT-proBNP after allogeneic HSCT [10-13] or after high dose cyclophosphamide [14]. We found a significant rise in the plasma NT-proBNP level one day after HSCT. This initial elevation in NT-proBNP levels might be a consequence of myocardial dysfunction caused by the conditioning regimen (TBI and/or chemotherapy), or previous ANT. It has been reported that a conditioning regimen causes an activation of endothelial cells and macrophages releasing inflammatory cytokines such as tumor necrosis factor alpha (TNF-α) or interleukins (IL) 1 and 6. There is increasing evidence that inflammatory cytokines may also play an important role in the pathogenesis of heart failure by inhibiting cardiac contractility, promoting myocardial hypertrophy and inducing cardiomyocyte apoptosis [15,16]. Elevated levels of NT-proBNP were found in 62,2% of patients even 14 days after HSCT. The same abnormalities were also found by Niwa et al (2002). Persistent elevations of NT-proBNP concentrations 30 days after HSCT were observed in 29,7% of patients, which might reflect subclinical cardiotoxicity.

Cardiac troponins have been defined as the biomarkers potentially useful for assessing minimal myocyte damage or loss of cell membrane integrity, and thus give structural information. The recently introduced highly sensitive troponin tests will expand the diagnostic potential to the detection of minor injuries of the heart and also higher cTnT levels, below the detection range of currently available assays may be considered as a marker of "end organ" cardiovascular damage [17,18]. For cardiotoxicity of anticancer drugs are typical low levels of cTnT. The majorities of these troponins are bound to actin and are released slowly. This characteristic, along with the slow clearance of troponins from the body permits the detection of not only acute damage but also of ongoing injury [19]. Baseline plasma hs-cTnT levels were elevated in 5 (13,5%) patients which might be associated with previous anthracycline exposure. Persistent low-level elevations at least 30 days after HSCT have been observed in our study in almost one third of patients, suggesting prolonged effects of chemotherapy or radiotherapy on the myofibrillar system of cardiomyocytes. Only some authors have demonstrated the role of cardiac troponins in detection of cardiotoxicity after allogeneic HSCT [13,20-22]. In fact, several studies have already shown continuous damage of chemotherapy or radiotherapy to the cardiac myofibrillar system [23-25]. In our study, levels of NT-proBNP showed positive correlation with hs-cTnT, which might indicate a relationship between the degree of structural myocyte damage and functional myocardial impairment. These observations were underscored by the echocardiographic studies which did reveal significant changes in systolic and diastolic parameters.

## Conclusions

Persistent elevations in cardiac biomarkers may reflect the presence of an underlying reduced functional myocardial reserve or reduced cardiac tolerance to cardiac stressors. Serial measurements of plasma NT-proBNP and hs-cTnT concentrations might be a useful tool for identification of patients at risk of developing cardiotoxicity after allogeneic HSCT and requiring cardiological follow up. Further studies are needed to clarify whether combination of both biomarkers improve detection of cardiotoxicity. Continued follow up is required to bring more insight into the predictive value of these biomarkers.

## Abbreviations

A: Peak flow velocity of late filling; ALL: Acute lymphoblastic leukemia; AML: Acute myeloid leukemia; ANT: Anthracyclines; CY: Cyclophosphamide; DT: E-wave deceleration time; E: Peak flow velocity of early filling; EF: Ejection fraction; GVHD: Graft-versus-host disease; HSCT: Hematopoietic stem cell transplantation; hs-cTnT: High sensitive cardiac troponin T; IVRT: Isovolumetric relaxation time; LV: Left ventricular; MDS: Myelodysplastic syndrome; NT-proBNP: N-terminal pro-B-type natriuretic peptide; TBI: Total body irradiation.

## Competing interests

The authors declare that they have no competing interests.

## Authors' contributions

LR designed the study, collected informations about patients, performed statistical analysis and drafted the manuscript, EB performed daily clinical evaluation of patients, revised the manuscript, MM revised the manuscript, JD performed echocardiography study and helped to revise the article, JG carried out biochemical studies and helped to revise the article, NL carried out biochemical studies, BM conceived the idea, revised the manuscript and supervised the study. All authors read and approved the final manuscript.
